# *In Vivo* Transplantation of Neurosphere-Like Bodies Derived from the Human Postnatal and Adult Enteric Nervous System: A Pilot Study

**DOI:** 10.1371/journal.pone.0093605

**Published:** 2014-04-03

**Authors:** Susan Hetz, Ali Acikgoez, Ulrike Voss, Karen Nieber, Heidrun Holland, Cindy Hegewald, Holger Till, Roman Metzger, Marco Metzger

**Affiliations:** 1 Translational Centre for Regenerative Medicine, University of Leipzig, Leipzig, Germany; 2 Fraunhofer Institute for Cell Therapy and Immunology, Clinic-oriented Therapy Assessment Unit, Leipzig, Germany; 3 Department of General and Visceral Surgery, St. George’s Hospital Leipzig, Leipzig, Germany; 4 Institute of Pharmacy, Pharmacology for Natural Sciences, University of Leipzig, Leipzig, Germany; 5 Department of Pediatric and Adolescent Surgery, Medical University of Graz, Graz, Austria; 6 Department of Pediatrics and Adolescent Medicine, Salzburg County Hospital, Salzburg, Austria; 7 Tissue Engineering and Regenerative Medicine, Fraunhofer IGB Project Group: Regenerative Technologies for Oncology, University Hospital Würzburg, Würzburg, Germany; Instituto Butantan, Brazil

## Abstract

Recent advances in the *in vitro* characterization of human adult enteric neural progenitor cells have opened new possibilities for cell-based therapies in gastrointestinal motility disorders. However, whether these cells are able to integrate within an *in vivo* gut environment is still unclear. In this study, we transplanted neural progenitor-containing neurosphere-like bodies (NLBs) in a mouse model of hypoganglionosis and analyzed cellular integration of NLB-derived cell types and functional improvement. NLBs were propagated from postnatal and adult human gut tissues. Cells were characterized by immunohistochemistry, quantitative PCR and subtelomere fluorescence *in situ* hybridization (FISH). For *in vivo* evaluation, the plexus of murine colon was damaged by the application of cationic surfactant benzalkonium chloride which was followed by the transplantation of NLBs in a fibrin matrix. After 4 weeks, grafted human cells were visualized by combined *in situ* hybridization (Alu) and immunohistochemistry (PGP9.5, GFAP, SMA). In addition, we determined nitric oxide synthase (NOS)-positive neurons and measured hypertrophic effects in the ENS and musculature. Contractility of treated guts was assessed in organ bath after electrical field stimulation. NLBs could be reproducibly generated without any signs of chromosomal alterations using subtelomere FISH. NLB-derived cells integrated within the host tissue and showed expected differentiated phenotypes i.e. enteric neurons, glia and smooth muscle-like cells following *in vivo* transplantation. Our data suggest biological effects of the transplanted NLB cells on tissue contractility, although robust statistical results could not be obtained due to the small sample size. Further, it is unclear, which of the NLB cell types including neural progenitors have direct restoring effects or, alternatively may act via ‘bystander’ mechanisms *in vivo*. Our findings provide further evidence that NLB transplantation can be considered as feasible tool to improve ENS function in a variety of gastrointestinal disorders.

## Introduction

The Enteric Nervous System (ENS) poses the largest and probably most complex division of the autonomic nervous system and mainly controls the principal activities of the gut including motility and secretion. Because of its important physiological role, disturbances of the ENS can lead to severe symptoms that, depending on the gut region, include dysphagia, gastro-esophageal reflux, delayed emptying of the stomach, abdominal pain and bloating, diarrhea, constipation, and fecal incontinence [1]. Symptoms are manifested in various diseases such as Hirschsprung’s disease (HSCR), achalasia, gastroparesis or chronic intestinal pseudo-obstruction [1], [2]. Although there has been much progress in diagnosis and therapy of these diseases, curative treatment options are still limited and the long-term outcome often remains unsatisfactory.

Multipotent neural stem and progenitor cells of the ENS have been identified as a promising cell source for the treatment of at least some ENS disorders, which may involve replacing or restoring neural components or, alternatively, stimulating endogenous regenerative response [3]–[11]. Recently, *in vitro* protocols have been developed which describe the propagation of mammalian neural stem and progenitor cells, mainly as neurosphere-like bodies (NLBs). These cells are capable of colonizing intestinal tissue and differentiating into neural subtypes demonstrated mostly *ex vivo* in organotypic tissue cultures [5], [8], [12]. There are few studies in which the biological potential of these cells have been evaluated *in vivo*. However, these studies were restricted to fetal or neonatal cell sources and often without proof of functional effects *in vivo* [13]–[17].

In this study, for the first time we determined the *in vivo* potential of human postnatal and adult ENS progenitors to functionally integrate into murine host tissue with disturbed ENS integrity. To induce damage of the ENS, we adapted a chemical treatment protocol using benzalkonium chloride (BAC), which was demonstrated to ablate the plexus of rodent colon tissue, dependent to the concentration and time of exposure [13], [18], [19]. Human enteric NLBs were transplanted into BAC-treated gut segments and tissue was analyzed histologically and functionally 4 weeks post-transplantation. Afterwards, within the gut wall, we could detect differentiated neurons, glial and smooth muscle-like cells derived from transplanted human cells. Although the total number of ENS cells was not significantly different, functional improvements in gut contractility were examined in organ bath studies. Therefore, our study provides first evidence that NLB transplantation is feasible to restore the motility function of guts with disturbed ENS integrity.

## Materials and Methods

### Ethics Statement

Full-thickness gut samples were obtained from patients undergoing gut resection surgery due to various diagnoses (Table 1). The study protocol was approved by the Ethical Committee of the Medical Faculty, University of Leipzig, Germany and written informed consent was obtained from the individuals to use ‘left-over’ parts of the resected biological material for research purposes (study approval number, Az. 066/2002). The data were analyzed anonymously and according to the principles expressed in the ‘Declaration of Helsinki’.

**Table 1:** Characteristics and analyses of human gut samples sorted with increasing patient age.

Sex	Age	Tissue,	Analyses						
		Diagnosis	OB	FISH	ISH	IHC	NOS	M	qPCR
m	0.1	rectum, at	nd	nd	nd	nd	nd	nd	6.3/0.05/18.4
f	0.5	jejunum, NEC	37	nd	19	614/227	51	65	nd
m	0.5	rectum, at	nd	nd	nd	nd	nd	nd	19.4/0.01/12.8
m	8	caecum, ap	nd	nd	nd	nd	nd	nd	0.05/0.04/31.8
f	14	ileum, IBD	53	nd	0	271/197	42	nd	nd
m	26	caecum, ap	42	nd	2	563/198	47	61	nd
m	40	ileum, ac	nd	nd	176	240/155	nd	107	nd
f	55	colon desc., p	11	nd	37	411/154	49	112	nd
m	57	colon sigm., ac	nd	nd	nd	nd	nd	52	nd
f	60	colon sigm., d	nd	nd	nd	nd	nd	nd	1.8/0.03/11.2
m	64	colon desc., ac	tp	nd	55	nd	nd	nd	nd
m	66	colon asc., ac	18	nd	0	nd	42	30	nd
m	70	colon asc., ac	nd	nd	nd	nd	nd	nd	0.7/0.01/7.7
m	72	colon desc., ac	nd	+	nd	nd	nd	nd	nd
m	73	colon sigm., ac	nd	nd	nd	nd	nd	nd	17.5/0.01/6.3
m	74	colon sigm., ac	nd	nd	nd	nd	nd	nd	4.3/0.10/11.5
m	74	colon desc., ac	nd	+	nd	nd	nd	nd	45.3/1.3/8.4
f	74	rectum, ac	nd	nd	nd	nd	nd	47	nd
f	76	colon desc., ac	nd	+	nd	nd	nd	nd	nd
f	82	colon sigm., ac	28	nd	6	234/189	37	32	nd
m	82	jejunum, ac	nd	nd	nd	nd	nd	68	nd
m	84	colon asc., ac	nd	nd	nd	nd	nd	nd	10.5/0.03/5.0
m	85	rectum, ac	nd	nd	7	nd	29	79	nd
f	95	colon desc., ac	tp	nd	53	nd	nd	130	nd

**Diagnosis:** ac, adenocarcinoma; ap, apendicitis; at, atresia; d, diverticulitis; IBD, inflammatory bowel disease; NEC, necrotizing enterocolitis; p, polyps.

**Analyses** (see ‘Methods’): **IHC**, immunohistochemistry (PGP9.5+ ganglia size in μm^2^/muscle thickness in μm); **ISH**, *in situ* hybridisation (human Alu+ cells per slice); **FISH**, fluorescence ISH (ToTelVysion DNA probe mixture); **M**, brightfield microscopy (sphere diameter in μm); **NOS**, diaphorase-NOS staining (% NOS+ of Fast Red plexus cells); **OB**, organ bath (% EFS of ACh contraction); **qPCR**, quantitative polymerase chain reaction (normalized 2^-ΔΔct^ values of p75/Ret/TPM); nd = not determined; tp = technical problems (data excluded).

The experimental animal *in vivo* protocols were approved by the Ethics Committee of the State Directorate Leipzig, Germany (study approval number, Az. 24-9168.11), and are in line with international guidelines for animal welfare.

### Generation and Culturing of Postnatal Enteric Progenitor Cells

Enteric human progenitor cells were isolated and cultured as described before [20]. Briefly, gut muscle strips were mechanically dissected and enzymaticaly digested using a collagenase/dispase enzyme solution (collagenase XI [750 U/mL; Sigma, Frickenhausen, Germany] and dispase II [250 μg/mL; Roche, Mannheim, Germany] in Hank’s balanced salt solution) for approximately 1 hour at 37°C under continuous rotating. Following trituration, the cell suspension was filtered through a 70 μm cell strainer and washed twice in Hank’s buffer. Cell pellet was resuspended in Dulbecco’s modified Eagle medium (DMEM)/F-12 medium (PAA, Coelbe, Germany) supplemented with penicillin (100 U/mL; PAA), streptomycin (100 mg/mL; PAA), L-glutamine (2 mmol/L; PAA), N2 (1∶100; Invitrogen, Karlsruhe, Germany), basic fibroblast growth factor (20 ng/mL; Peprotech, Hamburg, Germany), and epidermal growth factor (20 ng/mL; Peprotech). In all experiments, cells were initially seeded onto plastic dishes coated with fibronectin, ornithin and laminin (2 μg/cm^2^; Sigma). 50% of medium was supplemented with conditioned medium obtained from fast growing human fetal ENS (gut tissue obtained after elective pregnancy terminations) *in vitro* cell cultures. Every 2–3 days half of the medium was replaced with freshly prepared and conditioned medium. Cells were kept in culture for up to 21 days in a humidified incubator at 37°C, 5% CO_2_ and 2% O_2_ concentration. To induce cell differentiation, growth factors and conditioned medium were omitted from culture medium and 2% fetal calf serum and 200 μmol/L ascorbate-2-phosphate (Sigma) were added. The analysis of photographed NLBs and gut sections was estimated with ImageJ software (v1.38; National Institutes of Health, Bethesda, USA.).

### RNA Isolation and Quantitative PCR Analysis

Ribonucleic acid (RNA) isolation and quantitative polymerase chain reaction (qPCR) procedure was performed as described earlier [21]. Briefly, total RNA was isolated using an RNA Mini Kit and genomic desoxy-ribonucleic acid (gDNA) eliminator columns (Qiagen, Hilden, Germany). Purified RNA samples underwent reverse transcriptase reaction according to the supplier’s protocol (Superscript VILO cDNA Synthesis Kit; Invitrogen). Quantitative real-time PCR was performed in the ABI Prism 7500 Real Time PCR System (Applied Biosystems, Darmstadt, Germany) using the QuantiTect SYBR Green kit (Qiagen) and the following primer sets (forward/reverse primer; all from Invitrogen): p75 neurotrophin receptor [GenBank: NM_002507.3], a molecular marker expressed in enteric neural cells including stem and progenitor cells, (TGAGTGCTGCAAAGCCTGCAA/TCTCATCCTGGTAGTAG-CCGT; 230 bp), receptor tyrosine kinase (Ret) [GenBank: NM_020975.4], a neuronal marker including neural progenitors, (AGATTTCGGATTTCGGCTTGT/CCACAGC-AGGACACCAAAAGA; 161 bp), tropomyosin alpha [GenBank: NM_001018005.1], a smooth muscle cell marker, (TPM1; CTCGCAGAAGGAAGACAGATATGAG/TAGT-TACTGACCTCTCCGCAAACTC; 101 bp) and the housekeeping gene hypoxanthine phosphoribosyltransferase 1 (HPRT1) [GenBank: NM_000194.2] (TGAACGTCTTG-CTCGAGATGTG/CCAGCAGGTCAGCAAAGAATTT; 101 bp) to normalize gene expression data. Relative gene expression was determined with the 2(-Delta Delta C(T)) method. The quality of amplified PCR product was proved by melting curve analysis and agarose gel electrophoresis to estimate the size of each PCR product.

### Fluorescence *in situ* Hybridization and Cytogenetics

**Figure 1:**
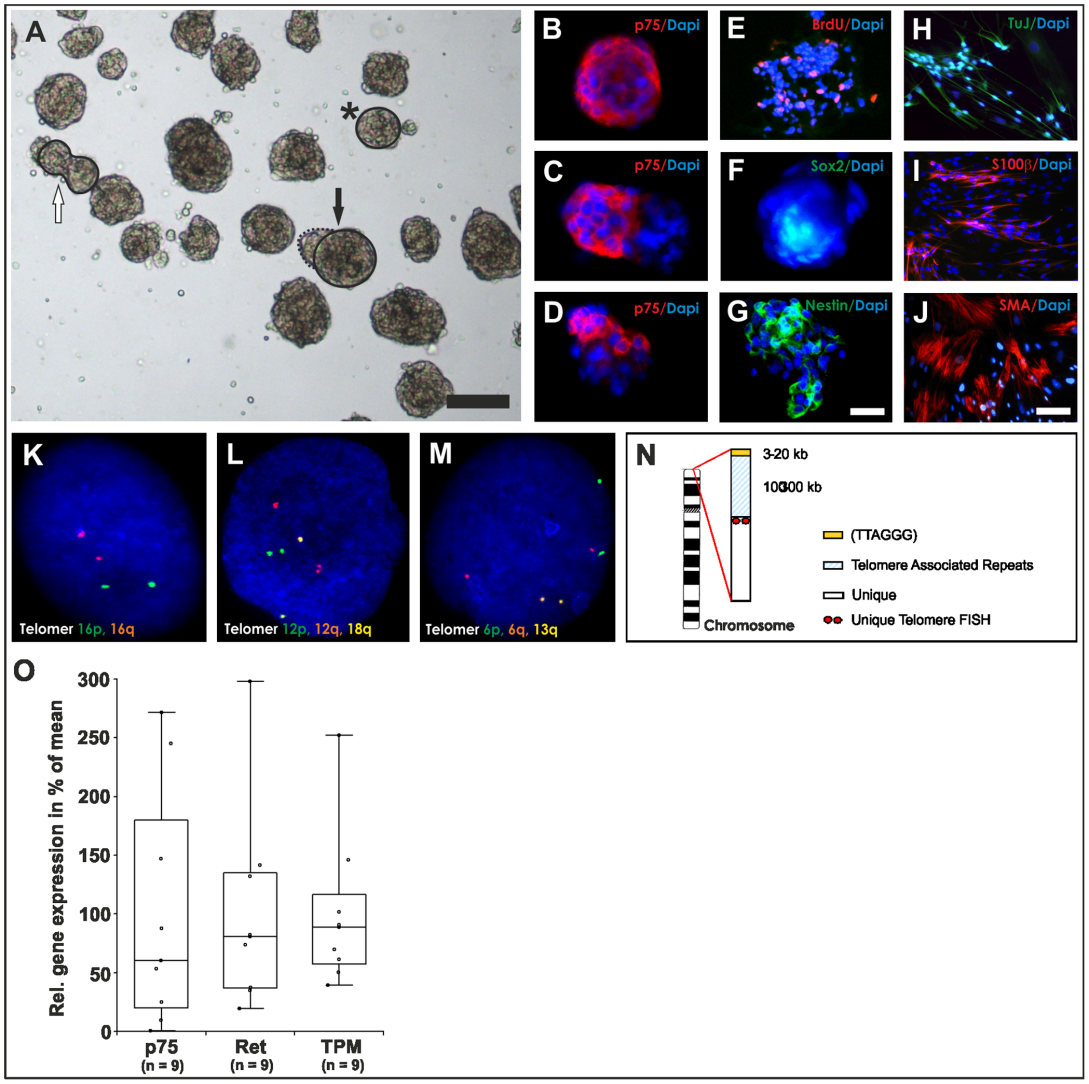
Characterization of multipotent neural progenitors isolated from human adult gut. (A) Bright-field view of representative adult enteric neurosphere-like bodies (NLBs) after 14 days *in vitro*. Free-floating NLBs could be observed with an estimated average number of 35 NLBs per preparation and a sphere diameter of 71.6 μm. Different NLB phenotypes include round- (star, comparable to B), ellipsoid- (white arrow, comparable to C) or irregular-shaped (black arrow, comparable to D) NLBs. (B–D) Depending to the individual NLB phenotype, different cellular compositions can be derived as shown for the ENS marker gene p75 in immunocytochemistry. (E–G) Proliferative NLB cells also incorporated BrdU and expressed previously described progenitor markers including Sox2 and Nestin. (H–J) After 2 weeks of differentiation NLBs built a monolayer composed of neuronal (TuJ), glial (S100β) and smooth muscle-like cells (SMA). (K–M) Molecular cytogenetic FISH analyses revealed no chromosomal alterations as representatively shown for three chromosomal interphase nuclei. (N) A schematic illustration of a chromosome is shown. In total, 15 different probe sets recognizing 41 different unique subtelomeric repeats were applied. (O) Efficacy and cellular distribution of NLBs strongly depends to the individual preparation, relying on age, amount and quality of tissue, as demonstrated by qPCR analysis for three representative marker genes: p75, Ret and tropomyosin (TPM). Scale bars represent in (A) 100 μm, (B–G) 25 μm, (H–J) 50 μm.

Genomic alterations of human enteric progenitors were evaluated by DNA fluorescence *in situ* hybridization (FISH) as described recently [22]. Briefly, after preparation of cell metaphases and interphases, cells were denatured, dehydrated and hybridized with multi-colour DNA FISH probe mixtures for 12–16 h at 37°C (ToTelVysionTM, Abbott, Wiesbaden, Germany). These probes bind to the sensitive subtelomeric chromosomal regions which are immediately adjacent to the long (TTAGGG)_n_ repeats and also contain repetitive stretches of DNA (Figure 1I). After washing, slides were air-dried for 20 min in the dark, incubated with 4′-6-Diamidino-2-phenylindole (DAPI) solution (125 μg/ml; Abbott, Wiesbaden, Germany) for 10 min at room temperature and coverslipped with Kaiser’s gelatine (Merck, Darmstadt, Germany).

### DNA in situ Hybridization

*In situ* hybridization (ISH) experiments were performed on 4% phosphate-buffered paraformaldehyde (PFA, PAA) fixed cryosections using a digoxigenin-labeled, 224–base pair antisense DNA probe corresponding to the consensus sequence of the human Alu sequence [23]. The ISH staining protocol was performed as described previously [20]. Color development was performed with Nuclear Fast Red salt substrate solution (Sigma) or nitro-blue tetrazolium (NBT)/5-bromo-4-chloro-3′-indolyphosphate (BCIP) (Roche) as described by the supplier. Alu-positive cells were counted microscopically (25×objective) from 4 non-consecutive longitudinal serial cryosections (∼15 μm×1.5 cm) of each animal (n = 10).

### Immunohistochemistry

Immunostaining was performed on cryosections of gut segments as described earlier [20]. Briefly, cryosections and NLBs were fixed in 4% PFA, rinsed in phosphate-buffered saline (PBS) (PAA) and blocked with blocking solution (PBS containing 0.3% Triton X-100 and 10% donkey serum, PAA) for 30 min. The following primary antibodies diluted in PBS with 0.1% Triton X-100 were applied overnight at 4°C: rabbit anti-p75 neurotrophin receptor (1∶250; p75, Promega, Mannheim, Germany), rabbit anti-S100β (1∶600; Dako, Hamburg, Germany), rabbit anti-smooth muscle actin (1∶400; SMA, AMS Biotechnology, Wiesbaden; Germany), rabbit anti-beta tubulin III (1∶2000; TuJ, Hiss Diagnostics, Freiburg, Germany), mouse anti-Nestin (1∶200; Millipore, Schwalbach, Germany), goat anti-SRY (sex determining region Y)-box 2 (1∶50, Sox2, Santa Cruz, Heidelberg, Germany), rabbit anti-glial fibrillary acidic protein (1∶600; GFAP, Dako, Hamburg, Germany) and rabbit anti-ubiquitin thiolesterase PGP9.5 (1∶500; PGP9.5, Millipore). After washing with PBS, the secondary antibodies donkey anti-rabbit IgG-Cy3, donkey anti-rabbit IgG-Alexa488, donkey anti-goat IgG-Alexa488 and donkey anti-mouse IgG-Alexa488 (1∶500, Invitrogen) were applied for 30 min at room temperature, additionally DAPI solution (1 μg/ml, Roth, Karlsruhe, Germany) in PBS with 0.1% Triton X-100 for 10 min and slides cover-slipped with Kaiser’s gelatin (Merck, Darmstadt, Germany). Image capturing was done on a Nikon inverted microscope (Nikon, Duesseldorf, Germany). All figures were assembled and annotated using Corel Draw (v11.633 software; Unterschleissheim, Germany) and ImageJ (v1.38; National Institutes of Health, Bethesda, USA.). For quantification of PGP9.5-staining, the area of fluorescent cells in the plexus myentericus region were measured microscopically (25×objective) from 4 non-consecutive longitudinal serial cryosections (15 μm×∼1–1.5 cm) of each animal (n = 6–7). Muscle thickness was measured on each microscopic field in the central position orthogonally from the outer muscle to the submucosal linings indicated by the characteristic nucleus stainings.

### BrdU Labeling

Cell proliferation was visualized by bromodeoxyuridine (BrdU) proliferation assay. BrdU (10 μmol/L) was added for 1 hour to the culture medium of proliferating spheres. Detection of ethanol-fixed BrdU-positive cells was carried out with the BrdU Detection Kit I (Roche, Mannheim, Germany).

### NADPH-diaphorase Staining

Nicotinamide adenine dinucleotide phosphate (NADPH)-diaphorase staining was performed as described earlier [24]. Briefly, 4% PFA fixed cryosections were incubated in the dark at 37°C for 30 min in a solution of 3 mL PBS, 1.5 mg NBT (Roche), 3 mg βNADPH and 1.5 μL Triton X-100 (Sigma). Samples were counterstained with Nuclear Fast Red solution (Vector Laboratories, Burlingame, USA) for 20 min at room temperature and cover-slipped using Kaiser’s gelatin. For analysis, Fast Red and nitric oxide synthase (NOS)-positive cells in the plexus myentericus region were counted microscopically (25×objective) from 4 non-consecutive longitudinal serial cryosections (15 μm×∼1–1.5 cm) of each animal (n = 5–7).

### Mouse Model and Transplantation of Human Enteric Progenitors *in vivo*

**Figure 2:**
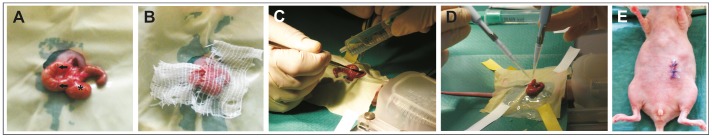
Experimental procedures of ENS ablation and cell transplantation. (A) Adult CD1 nude mice were anesthetized and the caecum was exposed through a 1 cm midline incision. (B) Cotton gauze soaked with 0.01% benzalkoniumchloride (BAC) was wrapped around ∼1.5 cm of proximal colon whereas the rest of the tissue was protected with cotton gauze soaked in NaCl solution. (C) After ∼20 min the BAC was thoroughly flushed away with NaCl solution. (D) 26 μl of fibrin/cell matrix, equally mixed of fibrinogen/cells and thrombin solution, was applied on the treated gut segment. (E) Guts were carefully placed back into the original *in vivo* position, the wound was sutured in two layers and finally sealed with medical wound glue.

8–12 weeks old (weight: ∼20–35 g) immunodeficient, female CD1 nude mice (Crl:CD1-Foxn1^nu^, Code 086/homozygous; Charles River, Sulzfeld, Germany) were used for transplantation experiments. Animals were anesthetized with 1–2% isoflurane/oxygen mixture (CP Pharma, Burgdorf, Germany), and the caecum was exposed through a 1 cm midline incision (Figure 2). Sterile cotton gauze soaked with 0.01% or 0.05% BAC in 0.9% sodium chloride (NaCl) solution (Delta Select, Munich, Germany) was wrapped around the proximal colon (length: ∼1.5 cm) whereas the rest of the tissue was protected with cotton gauze soaked in 0.9% NaCl solution. A drop of BAC solution was reapplied to the gauze every 2–3 minutes to prevent drying. After 20 minutes, the treated area and peritoneum were thoroughly flushed with NaCl solution. Prior transplantation, *in vitro* propagated NLBs were digested with accutase (PAA) for 5–10 minutes at 37°C (dissociation controlled microscopically), diluted in basal medium without any growth supplements up to 10 ml and centrifuged at 200 g. The pellet was then mechanically triturated in 3 ml fresh basal medium, centrifuged again and resuspended in 13 μl of basal medium. A mixture of 6.5 μl pre-warmed fibrinogen with 6.5 μl cell suspension in basal medium (∼10^5^ cells total) and 13 μl thrombin (Baxter, Munich, Germany) was equally applied on the treated gut segments using standard 20 μl pipettes in two consecutive steps. Control animals (i.e. BAC-treated only/negative control) received fibrin plus basal medium without cells. After solidification of the matrix (∼30 seconds), guts were carefully repositioned *in vivo* and the wound was sutured in two layers. Retacillin antibiotic (MIBE, Brehna, Germany) was intramuscularly injected directly after wound closure. A mixture of antibiotic (Baytril, Bayer, Leverkusen, Germany), analgetic (Metamizol, WDT, Garbsen, Germany) and glucose (Braun, Melsungen, Gemany) was given in the drinking water for the following 3 days according to the manufacturer’s recommendations. After 4 weeks post-transplantation, animals were killed via carbon dioxide (CO_2_) inhalation and cervical dislocation and resected tissues processed for functional and histological analyses (Table 1). In total, ten animals were transplanted with ten individual human donor cell samples.

### Electrical Field Stimulation and Isometric Muscle Recording

Functional evaluation of colonic contraction was measured in three experimental groups after four weeks post-transplantation: untreated control, BAC-treated and BAC-treated/cell-transplanted guts. Guts were carefully prepared, washed in Krebs buffer (Sigma) and connected to a force transducer via a string in an organ bath (TSE Systems, Bad Homburg, Germany). Tissue was pre-contracted and maintained in Krebs buffer gassed with carbogen (95% O_2_ und 5% CO_2_) at 37°C. Two control stimulations were performed via 1 μM acetylcholine (ACh, Sigma) at the beginning and end of each experiment. In between, two electrical field stimulations (EFS) (250 mA, 8 Hz, 5 ms pulse for 30 seconds) and one stimulation with 10^−5 ^M tetrodotoxin were applied (Biotrend, Cologne, Germany) to verify EFS-induced nerval activation. Following equilibration and control contraction, EFS-induced contraction was determined as percentage change from baseline muscle tone relative to the maximum acetylcholine contraction peak. For quantitative determination of EFS-induced contraction, intestinal gut segments from at least 6 mice per group were analyzed.

### Statistical Analysis

All data are expressed as mean and standard deviation (SD). Box-whisker plots show the 25^th^ and 75^th^ percentiles, the middle line represents the median, and the whiskers extend to the lowest and highest data points. Due to the limited and varying sample sizes, statistical significance was determined by nonparametric Kruskal-Wallis test and Fisher’s least significant difference (LSD) error protection as suitable alternative to analysis of variance (ANOVA) test. A P-value below 0.05 was considered to be statistically significant, a P-value between 0.05 and 0.15 was considered as moderate evidence for statistical significance with biological relevance.

## Results

### Characterization of Human Progenitors *in vitro*

With our experimental approach, we were able to reproducibly generate proliferating neural progenitors as neurosphere-like bodies (NLBs) from postnatal and adult human gut tissue. We observed a relatively high variability in growth efficiency and cellular composition dependent to age, amount and quality of gut samples. NLBs generation efficiency tend to be higher from younger donors compared to older donors considering same biopsy size and cultivation duration. After 2–3 weeks of *in vitro* culture, free-floating NLBs could be observed with an estimated average number of 35 NLBs per donor and a sphere diameter of 71.2^+^–32.8 μm (Figure 1A, n = 11 analyzed patients aged between 0.5 and 95 years, mean age: 56.3^+^–30.5 years). The total cell number counted from dissociated NLBs varied between 3×10^4^ and 2.2×10^5^ cells (mean: 9×10^4+^–7×10^4^ cells; n = 3). Expression of the neurotrophin receptor p75, a molecular marker also expressed in enteric neural progenitor cells, was found in 37.5^+^–12.1% of all NLB cells (n = 4) as analyzed by immunohistochemistry (Figure 1B–D). Other previously described neural progenitor markers were also present such as Sox2 or Nestin and cells incorporated BrdU indicating active proliferation (Fig. 1 E–G). The remaining non-neural cells stained for mesodermal markers such as smooth muscle actin, for instance (data not shown). Under differentiation conditions for 14 days NLBs rapidly built a monolayer, which included neuronal, glial and smooth muscle-like cells (Fig. 1H–J).

Interestingly, p75 was not homogenously expressed in the NLBs and three main types of NLBs could be identified: (i) round-shaped spheroids with compact appearance which uniformly expressed p75 (Figure 1A, star and Figure 1B) (ii) ellipsoid-shaped spheroids, probably generated by fusion of two smaller spheroids, mostly expressed less than 100% of p75 (Figure 1A, white arrow and Figure 1C) (iii) large irregular-shaped spheroids with darker appearance (p75-negative), partly fused with small, compact p75-positive stained cellular structures (Figure 1A, black arrow and Figure 1D). NLB types significantly varied between individual preparations, probably dependent to tissue integrity, which ultimately resulted in variations of the mRNA gene expression profile as shown for the three representative marker genes p75 (neural progenitors and differentiating cells), Ret (neuronal cells) and tropomyosin (TPM, smooth muscle-like cells) (Figure 1O; n = 9 analyzed patients aged between 2 month and 84 years, mean age: 52.1^+^–34.5 years). Interestingly, the neuronal (progenitor) marker gene Ret was significantly up-regulated (∼10-fold) in NLBs from older patients (60–84 years) compared to children (0–8 years), whereas no significant differences were observed with p75 or TPM (data not shown). Because p75 marks both, progenitors and differentiating neural cells (i.e. neurons and glia), changes in Ret expression suggests a relative increase in differentiated neuronal phenotypes in cell cultures obtained from older donors.

To further characterize NLBs, we performed a subtelomere FISH analysis to exclude DNA microdeletions or microduplications in the sensitive subtelomeric chromosomal regions (Figure 1N). We applied 15 probe mixtures detecting 41 specific subtelomere regions and found no indication of chromosomal instability after analyzing 80 interphase nuclei (n = 3, patients aged between 72 and 76 years) and according to the ‘International System for Human Cytogenetic Nomenclature’ 2009 [25]. In figure 1K–M, representative cell nuclei are shown demonstrating an inconspicuous FISH staining pattern.

### ENS Ablation and NLB Transplantation *in vivo*

**Figure 3:**
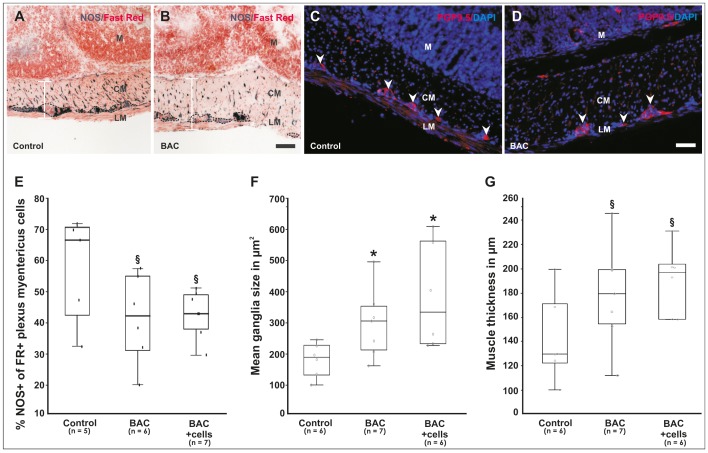
Alterations of the ENS four weeks post-operation. (A, B) Low-magnification bright-field view of NADPH-diaphorase staining demonstrates a visible and relative reduction (i.e. hypoganglionosis) of neuronal cells (shown as %NOS+ of Fast Red+ myenteric plexus cells, dotted lines) as well as neuronal fibers compared to untreated gut control tissues. Note that the musculature is thickened due to hypertrophic effects of BAC as indicated by the white bar. Nuclei are stained red with Nuclear Fast Red dye. (C, D) Compared to the control, hypertrophic effects are also detectable in the ENS as shown by immunohistochemistry for PGP9.5 (arrowheads). Nuclei were stained with DAPI (blue). (E) Quantification of NOS-positive cells in the myenteric plexus, (F) mean ganglia size indicated by PGP9.5 cytoplasmatic immunostaining, and (G) muscle thickness. As seen in box-whisker plots, damage of the ENS and muscle was still quantifiable four weeks after BAC-treatment, however in this analysis, no statistical significant effects were seen by additional cell transplantation compared to the BAC-only treated group. (A–D) Scale bars represent 100 μm. M = mucosa, CM = circular muscle, LM = longitudinal muscle. (E–G) *P<0.05 and 0.05< ^§^P<0.15 as compared to the untreated controls.

To constitute a small colonic segment with disturbed ENS function (i.e. hypoganglionosis), we applied the cationic surfactant benzalkonium chloride (BAC) (Figure 2). Dependent to the concentration and time of incubation, treatment with BAC has been shown to lead to transient enteric plexus ablation (i.e. hypoganglionosis) and hypertrophy as well as hypertrophy of the musculature. We have tested 2 different concentrations of BAC (0.01% and 0.05%) aiming to generate a moderate damage with minimal animal suffering and good survival of the recipient mice. The higher BAC concentration resulted in an already macroscopically visible damage of the treated gut segment (e.g. damaged vasculature) after 20 minutes of application (data not shown). Consequently, approximately 90% of all treated animals (n = 24) developed a severe intestinal obstruction and either died or were killed within the first 5 days to shorten animal suffering. After gut dissection, we noticed the constricted segments accompanied by extremely distended proximal colon/caecum as well as small intestine filled with abnormal amounts of condensed feces still visible in the surviving animals 4 weeks following operation. In contrast, 80% of animals (n = 28) treated with the lower BAC concentration survived the 4 weeks follow-up phase without any visible signs of suffering, weight-loss or macroscopically alterations of gut integrity. On a microscopic level, however we could still identify and quantify hypertrophic effects in the regenerating plexus structures and smooth musculature (Figure 3A–D, Figure 3F, G). In average, the mean size of ganglia, measured via the PGP9.5+ area, significantly increased from 180 μm^2^ (control) to 294 and 389 μm^2^ (BAC-treated groups without and with cells, respectively); 30–62 ganglia per animal were analyzed. In contrast, the mean cell number per ganglion, determined by Fast Red-positive cell number, was only moderately, but not significantly decreased after BAC-treatment. Between 250–450 total Fast Red+ cells per animal, organized as densely-packed ganglion structures, were counted. Interestingly, a relative decrease of NOS-positive neurons (in % of Fast Red-positive myenteric plexus cells), which represent the majority of plexus cells, and neuronal fibers was detectable in the BAC-treated animals (Figure 3B, E).

In order to evaluate the potential of NLB-derived cells to participate or promote *in vivo* plexus regeneration, we transplanted an average cell number of ∼10^5^ cells of unsorted neurosphere-derived cultures in a clinically approved fibrin matrix onto the complete serosal surface of the treated gut segments (Figure 2D). Interestingly, compared to the BAC-only group, in the (immuno-)histological analyses no statistically significant differences on both the number of NOS-neurons as well as the hypertrophy of the plexus (i.e. PGP9.5+ mean ganglia size) and smooth musculature were observed (Figure 3E–G). Noteworthy, if considering only the subgroup of older donors (>40 years), the hypertrophic effects tend to be less pronounced compared to the younger donor group suggesting age-dependent effects. Due to the small number of patients, however this could not be statistically ensured.

### Integration of Human NLBs *in vivo*

**Figure 4:**
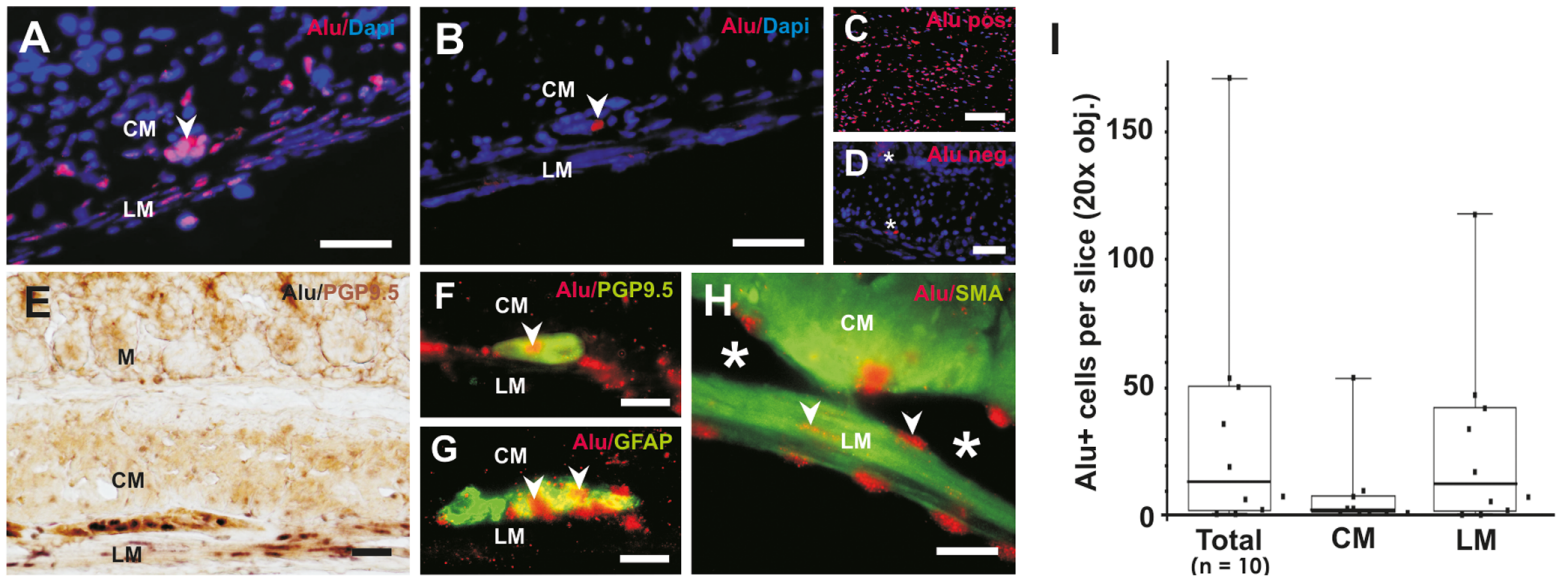
Integration of transplanted human NLBs *in vivo*. (A) Example of *in situ* hybridization (ISH) for human-specific Alu sequence with good cell integration within the gut musculature. Note that ganglia-like structures can be detectedat the expected location between the circular and longitudinal muscle layer (arrowhead). (B) Representative example for poor cell integration i.e. only a single cell was detected in this microscopic view (arrowhead). (C) Alu-ISH on human control gut tissue demonstrates the specifity of Alu probe for human cells. (D) ISH with Alu probe on mouse tissue served as negative control. Note, that there is some unspecific background staining in the cytoplasm and extracellular matrix, which does not co-localize with Dapi+ nuclei (stars). (E) Alu-ISH was combined with immunohistochemistry (IHC) to demonstrate *in vivo* differentiation state of neuronal cells between the longitudinal and circular muscle layers (Alu, black nuclei and PGP9.5, brown cytoplasmic staining). (F–H) Fluorescence ISH for Alu combined with IHC in a higher magnification (Alu-ISH, red nuclei and IHC, green cytoplasma) (F) A PGP9.5/Alu co-stained human cell has been integrated in a small ganglion structure (arrowhead). (G) Differentiated human glial cells within a ganglion as shown by Alu/GFAP-co-staining (arrowheads). (H) Most of the transplanted cells (arrowheads) were found around ganglion structures (stars) in the smooth muscle layers as shown by Alu/SMA-co-staining. (I) Variability of human cell integration was quantified and demonstrated as box-whisker plots. Most cells were found within the longitudinal muscle layer. Scale bars in (A–D) 100 μm, (E) 50 μm, (F–H) 200 μm. M = mucosa, CM = circular muscle, LM = longitudinal muscle.

To clarify the transplanted cell fate i.e. whether and where the transplanted human progenitors have been integrated within the host tissue, we performed DNA *in situ* hybridization (ISH) using a probe for the highly repetitive Alu sequence that specifically detects human cell nuclei as proved in human and mouse control tissues (Figure 4C, D). In our experiments, we detected Alu-positive human cells in 8 of 10 transplanted animals, although with high variability (Figure 4A, B, I; n = 10 animals ranging from 0 to 176 Alu+ cells/slice, mean: 35.2^+^–53.7 cells/slice). 5 of the 8 animals revealed relative high numbers of integrated human cells (∼20–180 cells/slice), although the estimated total number of surviving cells in the treated gut segment is far less than the initial transplanted cell number suggesting relatively moderate cell survival and/or integration. Noteworthy, there seem to be slight age-dependent effects of donors with respect to an increased extent of cell integration; however, the number of analyzed patients was too low to apply any robust statistics. Most of the human cells were found within the longitudinal muscle (79%), the remaining cells in the inner circular muscle region (21%). Interestingly, we also discovered cells clustering in the known plexus region suggesting either neo-ganglia formation or integration of transplanted neural cells into existing/regenerating plexus structures (Figure 4A, arrowhead). This observation could also be verified by co-immunostaining with characteristic ENS differentiation markers, such as PGP9.5 to identify neurons (Figure 4E, F) and GFAP for glial cells (Figure 4G) indicating the presence and survival of neural cells *in vivo*. However, the majority of cells were not found within ganglia structures, but rather as single cells located in the smooth muscle layers or in close contact to ganglia. Co-staining for SMA verified the presence of non-neural phenotypes such as smooth muscle-like cells (Figure 4H). Unfortunately, due to technical reasons a quantification of double-stained human cells was not applicable since all used antibodies, established for *in situ* hybridization and immunohistochemistry, are cytoplasmic markers, and therefore not suitable for a precise allocation.

### Analysis of Gut Contractility

**Figure 5:**
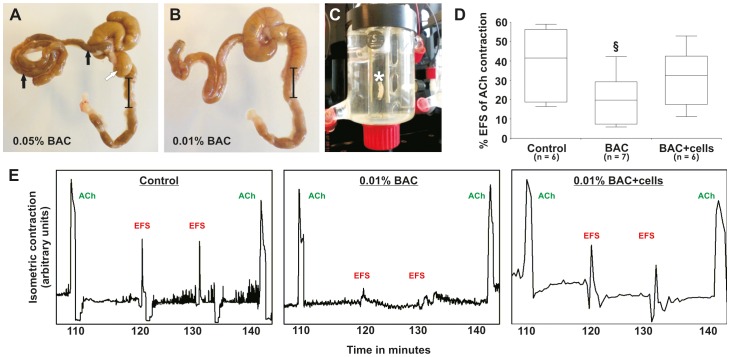
Macroscopic evaluation and organ bath analysis of transplanted gut tissues. (A, B) Concentration-dependent severity of gut/ENS damage after BAC-treatment is visible in resected gut tissues. Higher concentrations resulted in severe obstruction proximal to the treated gut segments indicated by condensed feces and tissue dilatations (arrows) leading to death of the majority of animals within few days. Lower BAC-concentrations instead led to a moderate damage of the gut tissue with good animal survival. (C) Segments treated with 0.01% BAC (black bar, with and without cell transplantation) and control gut tissues (i.e. normal gut) were placed in the organ bath between two electrodes (star) for further analysis of EFS-induced gut contractility. (D) Quantification of EFS-induced isometric contractility relative to the Acetylcholine (ACh)-induced control contraction is shown in box-whisker plots. The tissue damage caused by BAC (^§^P = 0.06) could be partly abolished after cell transplantation (P = 0.43) compared to control tissues. (E) Representative plots showing the ACh control peaks at the beginning and end of each measurement. After EFS application, contractions were particularly diminished in the BAC-only treatment group, which could be partly restored after cell transplantation.

According to our ISH results, there was the possibility that we might able to detect beneficial effects of human cell transplantation on a functional level. Therefore, we first demonstrated that BAC could cause detrimental effects on gut contractility after the 4 weeks follow-up as a consequence of the ENS damage seen in histology. Resected gut segments (Figure 5A–C) were placed into an organ bath filled with physiological Krebs solution and electrical field stimulations (EFS) was performed to assess neurally mediated contractions of the smooth musculature. Acetycholine (ACh)-induced contractions were included before and after EFS and served as reference control (Figure 5E). For unknown reasons, 5 of 24 analyzed guts (2×control, 1×BAC, 2×BAC +cells) showed no reaction, neither to ACh nor to EFS, probably due to technical problems and therefore could not be included in the analysis. In the remaining guts, BAC-treatment caused a notable ∼50% inhibition of contractile response after EFS (Figure 5D; n = 7, P = 0.06 vs. controls), although the variability of results was relatively high, which resulted in a moderate statistical P-value. In contrast, this inhibitory effects on contractility was reduced in the transplantation group (Figure 5D; n = 6, P = 0.43 vs. controls), which was even more distinct if only the younger donors were considered. The functional data therefore suggest that NLB transplantation indeed may have physiological effects, even if not clearly reflected on a histological level.

## Discussion

Several studies in mammals have confirmed the presence of multipotent adult stem and progenitor cells in ENS *in vitro* and *in vivo*. This was shown in age-dependent biomarker studies [26]–[29], ENS ablation *in vivo* models [18], [30], [31] and numerous *in vitro* ENS stem cell studies [4], [17], [28], [32]–[34]. A more recent *in vivo* approach in adult mice additionally specified enteric neurogenesis taking place within stem cell niches between the myenteric plexus and the longitudinal muscle layer [35]. In comparison to animal studies first *in vitro* approaches could also successfully demonstrate stem- and progenitor cells in human gut, mainly propagated as NLBs [5], [8], [12]. NLBs were isolated from early postnatal full-thickness gut samples and showed a heterogeneous expression of ENS progenitor markers (Sox10, p75, Ki67) as well as mature neuronal subtype markers especially after induction of differentiation [36]–[38]. More recently, we established protocols for the propagation of NLBs from human adult gut samples (aged up to 89 years), which could be differentiated into electrophysiological active neurons *in vitro* [20]. In the present study, we modified the existing protocols by adding conditioned medium from human fetal cultures instead of murine fetal cultures and NLBs were kept under hypoxic culture conditions (2% O_2_) instead of normoxic conditions (∼20% O_2_), which revealed further beneficial effects on NLB expansion [21]. Our cytogenetic analysis (i.e. subtelomeric FISH) revealed no detrimental effects of our culture protocol on chromosomal stability. Analyzing chromosomal aberrations is an important safety issue for future clinical applications of enteric neural cell-based therapies. It is also worth noting that multipotent human neural progenitors can principally be isolated from mucosal biopsies, which represents a practical advance towards the development of future cell-therapeutic approaches [39]. After injection into aganglionic gut explant cultures, postnatal and adult human progenitors have been shown to differentiate into neurons and glia and could partly improve gut functionality *in vitro* [20], [38].

Overall, these exciting progresses in the field of ENS stem cell research have suggested alternative therapeutic, autologous cell-based options in the treatment of gastrointestinal motility disorders, knowing that the current are unsatisfactory and rather palliative than curative [3], [6], [40], [41]. However, a critical step for the further translation of this concept into clinical studies would be pre-clinical animal *in vivo* studies. There are few studies in which ENS progenitors, isolated either from fetal or early postnatal mammalian gut, have been transplanted in rodents leading to a significant proportion of differentiated cells within the host tissue [13]–[17]. Two of the studies also performed functional examinations demonstrating recovery of intestinal motility [14], [17].

In contrast to previous studies, for the first time we evaluated the biological *in vivo* potential of human adult progenitors and included functional analyses. The high variance observed in the propagation of NLBs *in vitro* seems strongly dependent to the quality, amount and age of the individual gut biopsy and has been noticed before [28], [32], [39]. In addition, NLBs contained a relatively heterogeneous mixture of progenitors, demonstrated by ∼40% p75-immunopositive cells and further differentiated cells (i.e. neurons, glia, mesenchymal cell derivatives). This phenomenon has also been described for NLBs from the central nervous system before and poses a practical problem for standardized approaches [42], [43]. However, despite knowing the associated disadvantages of NLBs, but in light of this first scientific proof-of-concept study, we continued to use this well-established culture technology and only considered the total cell numbers because (i) no standardized protocols for pure human monolayer ENS stem cell cultures have been established so far, (ii) no exclusive cell surface biomarkers allowing to apply sorting techniques for human ENS progenitors are available, (iii) NLB generation is the state-of-the-art technology to generate a minimum ENS cell mass necessary to perform any *in vivo* experiment, and (iv) NLBs represent an approximated *in vivo* ENS niche which could thoroughly have beneficial effects for survival and functional integration of transplanted cells. However, there is no doubt that we have to further improve and standardize the existing protocols for future studies that we can finally provide acceptable protocols for clinical studies. This includes particularly the development of efficient scale-up protocols in progenitor cell culture and subsequent cell sorting strategies.

A second question also addressed in our study was to find a feasible cell delivery method to the site of transplantation. The *in vivo* studies mentioned above either injected cells locally into the gut or via the intraperitoneal route. Both strategies certainly have their advantages and were successful in the sense that cells, at least in parts, survived and integrated into the host tissue. However, the intramural, technically quite challenging injection method needs to be done repeatedly to cover larger areas of denervated gut segments and bears the obvious risk of life-threatening gut perforation. Additionally, only small volumes of concentrated cell suspensions can be injected at one time, which might be insufficient to cover larger areas (e.g. due to cell clumping and limited spatial distribution). Intraperitoneal injection of cells is a quick procedure and certainly may cover larger tissue areas, however, in a rather unspecific way which eventually poses a considerable safety risk in clinical applications. In addition, the colon seems not be susceptible for this technique [15]. As an alternative approach, we therefore tested a biodegradable fibrin matrix which has been clinically approved for several years and is available as sterile-packed and ready-to-use spraying device. With this technique, we observed notable cell integration in 40% of all transplanted animals, only 20% of all animal showed no cell integration in histology. Besides the positive regulatory aspect, this technique also combines scientific advantages of the other mentioned delivery routes in the respect that it is a quick procedure and covers large tissue areas at the same time. Furthermore, important stimuli such as cell growth factors, survival factors or immune-modulatory drugs could be additionally immobilized in the matrix in future studies. Nevertheless, also with this technique, cells still need to home from the matrix into the gut tissue. This leads to remaining risks that parts of the matrix are either sheared during re-positioning of the gut after surgery or due to normal gut peristalsis and potentially settle into other target tissues. To prove this, time-dependent *in vivo* tracking and safety studies would be interesting and necessary.

In order to induce ENS dysfunction *in vivo,* we used the chemical benzalkoniumchloride (BAC)-induced denervation model, which has been previously described in rodents [14], [19], [44], [45]. In contrast to available genetic animal models (e.g. Sox10^−/−^, Ret^−/−^), this model allows the induction of dysfunctional innervation in a well-defined segment of adult gut. In this respect, it is interesting to note, that ENS donor cells prefer to migrate into aneural gut segments than in tissues containing an intact ENS, as shown in a previous study [46]. Due to transplantation of human cells in this study, immunodeficient CD1 nude mice were used to prevent immune rejection. The higher sensitivity to exogenous influences, caused by the lack of thymus, prompted us to use a relatively low BAC concentration (0.05%) which has been published for Balb/c wildtype mice before [18]. However, we experienced high animal mortality with this concentration, mainly due to severe intestinal obstruction. In contrast, further reduction to 0.01% BAC finally resulted in good animal survival with still detectable damaging effects in histology (i.e. ENS hypoganglionosis, neuronal and muscular hypertrophy) as well as organ bath experiments (i.e. disturbed contractility) after 4 weeks follow-up. Interestingly, whereas little or no differences in histology were seen between the BAC-treated group and the BAC-treated/cell-transplanted group, notable biological effects in the functional evaluation were detected, although with moderate statistical P-values due to the relatively low number of animals included in this pilot study. However, our results suggest that the transplanted NLB cells did indeed have notable biological effects on the integrative neural activity either by replacing dead or dying ENS cells or by acting as ‘bystander cells’ stimulating the intrinsic regenerative response. In this respect, it is worth noting, that most of the transplanted cells were outside of ganglia and negative for pan-neural differentiation markers (PGP9.5, GFAP) supporting mainly the ‘bystander theory’ and data from a recently published *in vivo* study in rats [15]. Furthermore, there is a possibility that these cells are still undifferentiated stem cells residing in the germinal niches between the myenteric plexus and longitudinal muscle in adult mice [35]. In future studies, it would be of great interest to investigate this theory by doing cell fate mapping studies beyond the 4 week time point. Furthermore, large-scale and large animal studies will be necessary to confirm our first observations. Nevertheless, a small, but critical number of cells seem to be capable to restore parts of gut function which is promising considering the clinical need for treatments of rather larger areas of diseased gut tissue.

## Conclusions

In conclusion, we could demonstrate the reproducible, serum-free propagation of human adult enteric progenitors as NLBs with no signs of chromosomal alterations using subtelomere FISH. These NLBs, suspended in a clinical-approved fibrin matrix, were able to integrate and survive following *in vivo* transplantation in murine gut. Notable biological effects of transplanted cells on gut contractility were observed in an immunodeficient mouse model for hypoganglionosis. Thus, our pilot study provides the first *in vivo* validation of human adult enteric NLBs in an animal model and support the idea that these cells may be a promising, almost unlimited source for novel cell-based therapeutics to cure patients with gut motility disorders.

## References

[1] De GR, Guerrini S, Barbara G, Cremon C, Stanghellini V, et al (2004) New insights into human enteric neuropathies. Neurogastroenterol Motil 16 Suppl 1 143–7 143–147. 10.1111/j.1743-3150.2004.00491.x 15066021

[2] Di NG, Blandizzi C, Volta U, Colucci R, Stanghellini V, et al (2008) Review article: molecular, pathological and therapeutic features of human enteric neuropathies. Aliment Pharmacol Ther 28: 25–42. 18410560 10.1111/j.1365-2036.2008.03707.x

[3] Micci MA, Pasricha PJ (2007) Neural stem cells for the treatment of disorders of the enteric nervous system: strategies and challenges. Dev Dyn 236: 33–43 10.1002/dvdy.20975 [doi] 17029286

[4] Hotta R, Natarajan D, Burns AJ, Thapar N (2011) Stem cells for GI motility disorders. Curr Opin Pharmacol 11: 617–623. S1471-4892(11)00171-8 [pii]; 10.1016/j.coph.2011.09.004 [doi]. 10.1016/j.coph.2011.09.004 22056114

[5] Kulkarni S, Becker L, Pasricha PJ (2012) Stem cell transplantation in neurodegenerative disorders of the gastrointestinal tract: future or fiction? Gut 61: 613–621. gut.2010.235614 [pii]; 10.1136/gut.2010.235614 [doi]. 10.1136/gut.2010.235614 PMC4119942 21816959

[6] Hotta R, Natarajan D, Thapar N (2009) Potential of cell therapy to treat pediatric motility disorders. Semin Pediatr Surg 18: 263–273. S1055-8586(09)00046-8 [pii]; 10.1053/j.sempedsurg.2009.07.008 [doi].

[7] Gershon MD (2007) Transplanting the enteric nervous system: a step closer to treatment for aganglionosis. Gut 56: 459–461. 56/4/459 [pii]; 10.1136/gut.2006.107748 [doi]. 10.1136/gut.2006.107748 PMC1856867 17369379

[8] Schafer KH, Micci MA, Pasricha PJ (2009) Neural stem cell transplantation in the enteric nervous system: roadmaps and roadblocks. Neurogastroenterol Motil 21: 103–112. NMO1257 [pii]; 10.1111/j.1365-2982.2008.01257.x [doi]. 10.1111/j.1365-2982.2008.01257.x 19215588

[9] Estrada-Mondaca S, Carreon-Rodriguez A, Belkind-Gerson J (2007) Biology of the adult enteric neural stem cell. Dev Dyn 236: 20–32 10.1002/dvdy.20954 [doi] 16972279

[10] Burns AJ, Pasricha PJ, Young HM (2004) Enteric neural crest-derived cells and neural stem cells: biology and therapeutic potential. Neurogastroenterol Motil 16 Suppl 13–7 10.1111/j.1743-3150.2004.00466.x [doi];NMO466 [pii] 15065996

[11] Young HM (2005) Neural stem cell therapy and gastrointestinal biology. Gastroenterology 129: 2092–2095. S0016-5085(05)02192-X [pii]; 10.1053/j.gastro.2005.10.033 [doi]. 10.1053/j.gastro.2005.10.033 16344074

[12] Metzger M (2010) Neurogenesis in the enteric nervous system. Arch Ital Biol 148: 73–83. 20830970

[13] Geisbauer CL, Wu BM, Dunn JC (2012) Transplantation of enteric cells into the aganglionic rodent small intestines. J Surg Res 176: 20–28. S0022-4804(11)00453-7 [pii]; 10.1016/j.jss.2011.05.014 [doi]. 10.1016/j.jss.2011.05.014 21704327

[14] Pan WK, Zheng BJ, Gao Y, Qin H, Liu Y (2011) Transplantation of neonatal gut neural crest progenitors reconstructs ganglionic function in benzalkonium chloride-treated homogenic rat colon. J Surg Res 167: e221-e230. S0022-4804(11)00013-8 [pii]; 10.1016/j.jss.2011.01.016 [doi]. 10.1016/j.jss.2011.01.016 21392806

[15] Tsai YH, Murakami N, Gariepy CE (2011) Postnatal intestinal engraftment of prospectively selected enteric neural crest stem cells in a rat model of Hirschsprung disease. Neurogastroenterol Motil 23: 362–369 10.1111/j.1365-2982.2010.01656.x [doi] 21199176 PMC3105196

[16] Martucciello G, Brizzolara A, Favre A, Lombardi L, Bocciardi R, et al (2007) Neural crest neuroblasts can colonise aganglionic and ganglionic gut in vivo. Eur J Pediatr Surg 17: 34–40 10.1055/s-2007-964952 [doi] 17407019

[17] Anitha M, Joseph I, Ding X, Torre ER, Sawchuk MA, et al (2008) Characterization of fetal and postnatal enteric neuronal cell lines with improvement in intestinal neural function. Gastroenterology 134: 1424–1435. 18471518 10.1053/j.gastro.2008.02.018 PMC2612783

[18] Hanani M, Ledder O, Yutkin V, Abu-Dalu R, Huang TY, et al (2003) Regeneration of myenteric plexus in the mouse colon after experimental denervation with benzalkonium chloride. J Comp Neurol 462: 315–327. 12794735 10.1002/cne.10721

[19] Dong YL, Liu W, Gao YM, Wu RD, Zhang YH, et al (2008) Neural stem cell transplantation rescues rectum function in the aganglionic rat. Transplant Proc 40: 3646–3652. 19100458 10.1016/j.transproceed.2008.06.107

[20] Metzger M, Bareiss PM, Danker T, Wagner S, Hennenlotter J, et al (2009) Expansion and differentiation of neural progenitors derived from the human adult enteric nervous system. Gastroenterology 137: 2063–2073. 19549531 10.1053/j.gastro.2009.06.038

[21] Hegewald C, Alt R, Hetz S, Cross M, Acikgoez A, et al (2011) Reduced oxygen stress promotes propagation of murine postnatal enteric neural progenitors in vitro. Neurogastroenterol Motil 23: e412–e424 10.1111/j.1365-2982.2011.01761.x [doi] 21815967

[22] Holland H, Ahnert P, Koschny R, Kirsten H, Bauer M, et al. (2012) Detection of novel genomic aberrations in anaplastic astrocytomas by GTG-banding, SKY, locus-specific FISH, and high density SNP-array. Pathol Res Pract 208: 325–330. S0344-0338(12)00102-1 [pii]; 10.1016/j.prp.2012.03.010 [doi]. 10.1016/j.prp.2012.03.010 22575435

[23] Just L, Timmer M, Tinius J, Stahl F, Deiwick A, et al. (2003) Identification of human cells in brain xenografts and in neural co-cultures of rat by in situ hybridisation with Alu probe. J Neurosci Methods 126: 69–77. S0165027003000657 [pii]. 10.1016/s0165-0270(03)00065-7 12788503

[24] Wallace AS, Barlow AJ, Navaratne L, Delalande JM, Tauszig-Delamasure S, et al. (2009) Inhibition of cell death results in hyperganglionosis: implications for enteric nervous system development. Neurogastroenterol Motil 21: 768-e49. NMO1309 [pii]; 10.1111/j.1365-2982.2009.01309.x [doi]. 10.1111/j.1365-2982.2009.01309.x 19400926

[25] Brothman AR, Persons DL, Shaffer LG (2009) Nomenclature evolution: Changes in the ISCN from the 2005 to the 2009 edition. Cytogenet Genome Res 127: 1–4. 000279442 [pii]; 10.1159/000279442 [doi]. 10.1159/000279442 20110655

[26] Young HM, Bergner AJ, Muller T (2003) Acquisition of neuronal and glial markers by neural crest-derived cells in the mouse intestine. J Comp Neurol 456: 1–11. 12508309 10.1002/cne.10448

[27] Heanue TA, Pachnis V (2011) Prospective identification and isolation of enteric nervous system progenitors using Sox2. Stem Cells 29: 128–140 10.1002/stem.557 [doi] 21280162 PMC3059409

[28] Kruger GM, Mosher JT, Bixby S, Joseph N, Iwashita T, et al (2002) Neural crest stem cells persist in the adult gut but undergo changes in self-renewal, neuronal subtype potential, and factor responsiveness. Neuron 35: 657–669. 12194866 10.1016/s0896-6273(02)00827-9 PMC2728576

[29] Azan G, Low WC, Wendelschafer-Crabb G, Ikramuddin S, Kennedy WR (2011) Evidence for neural progenitor cells in the human adult enteric nervous system. Cell Tissue Res 344: 217–225 10.1007/s00441-011-1130-9 [doi] 21369860

[30] Cracco C, Filogamo G (1997) Neuronal and non-neuronal plasticity in the rat following myenteric denervation. Adv Exp Med Biol 429 159–69 159–169. 10.1007/978-1-4757-9551-6_12 9413573

[31] Poli E, Lazzaretti M, Grandi D, Pozzoli C, Coruzzi G (2001) Morphological and functional alterations of the myenteric plexus in rats with TNBS-induced colitis. Neurochem Res 26: 1085–1093. 11699935 10.1023/a:1012313424144

[32] Bondurand N, Natarajan D, Thapar N, Atkins C, Pachnis V (2003) Neuron and glia generating progenitors of the mammalian enteric nervous system isolated from foetal and postnatal gut cultures. Development 130: 6387–6400. 14623827 10.1242/dev.00857

[33] Silva AT, Wardhaugh T, Dolatshad NF, Jones S, Saffrey MJ (2008) Neural progenitors from isolated postnatal rat myenteric ganglia: expansion as neurospheres and differentiation in vitro. Brain Res 1218: 47–53. S0006-8993(08)00979-7 [pii]; 10.1016/j.brainres.2008.04.051 [doi]. 10.1016/j.brainres.2008.04.051 18514173

[34] Suarez-Rodriguez R, Belkind-Gerson J (2004) Cultured nestin-positive cells from postnatal mouse small bowel differentiate ex vivo into neurons, glia, and smooth muscle. Stem Cells 22: 1373–1385. 15579654 10.1634/stemcells.2003-0049

[35] Liu MT, Kuan YH, Wang J, Hen R, Gershon MD (2009) 5-HT4 receptor-mediated neuroprotection and neurogenesis in the enteric nervous system of adult mice. J Neurosci 29: 9683–9699. 19657021 10.1523/JNEUROSCI.1145-09.2009 PMC2749879

[36] Rauch U, Hansgen A, Hagl C, Holland-Cunz S, Schafer KH (2006) Isolation and cultivation of neuronal precursor cells from the developing human enteric nervous system as a tool for cell therapy in dysganglionosis. Int J Colorectal Dis 21: 554–559. 16267668 10.1007/s00384-005-0051-z

[37] Almond S, Lindley RM, Kenny SE, Connell MG, Edgar DH (2007) Characterisation and transplantation of enteric nervous system progenitor cells. Gut 56: 489–496. 16973717 10.1136/gut.2006.094565 PMC1856871

[38] Lindley RM, Hawcutt DB, Connell MG, Almond SN, Vannucchi MG, et al (2008) Human and mouse enteric nervous system neurosphere transplants regulate the function of aganglionic embryonic distal colon. Gastroenterology 135: 205–216. 18515088 10.1053/j.gastro.2008.03.035

[39] Metzger M, Caldwell C, Barlow AJ, Burns AJ, Thapar N (2009) Enteric nervous system stem cells derived from human gut mucosa for the treatment of aganglionic gut disorders. Gastroenterology 136: 2214–2225. 19505425 10.1053/j.gastro.2009.02.048

[40] Thapar N (2009) New frontiers in the treatment of Hirschsprung disease. J Pediatr Gastroenterol Nutr 48 Suppl 2 S92–4 S92–S94. 10.1097/MPG.0b013e3181a15d62 19300137

[41] Amiel J, Lyonnet S (2001) Hirschsprung disease, associated syndromes, and genetics: a review. J Med Genet 38: 729–739. 11694544 10.1136/jmg.38.11.729 PMC1734759

[42] Suslov ON, Kukekov VG, Ignatova TN, Steindler DA (2002) Neural stem cell heterogeneity demonstrated by molecular phenotyping of clonal neurospheres. Proc Natl Acad Sci U S A 99: 14506–14511 10.1073/pnas.212525299 [doi]; 212525299 [pii] 12381788 PMC137913

[43] Singec I, Knoth R, Meyer RP, Maciaczyk J, Volk B, et al. (2006) Defining the actual sensitivity and specificity of the neurosphere assay in stem cell biology. Nat Methods 3: 801–806. nmeth926 [pii]; 10.1038/nmeth926 [doi]. 10.1038/nmeth926 16990812

[44] Hanani M, Ledder O, Yutkin V, Abu-Dalu R, Huang TY, et al (2003) Regeneration of myenteric plexus in the mouse colon after experimental denervation with benzalkonium chloride. J Comp Neurol 462: 315–327 10.1002/cne.10721 [doi] 12794735

[45] Liu W, Wu RD, Dong YL, Gao YM (2007) Neuroepithelial stem cells differentiate into neuronal phenotypes and improve intestinal motility recovery after transplantation in the aganglionic colon of the rat. Neurogastroenterol Motil 19: 1001–1009. 17973630 10.1111/j.1365-2982.2007.00981.x

[46] Hotta R, Anderson RB, Kobayashi K, Newgreen DF, Young HM (2010) Effects of tissue age, presence of neurones and endothelin-3 on the ability of enteric neurone precursors to colonize recipient gut: implications for cell-based therapies. Neurogastroenterol Motil 22: 331-e86. NMO1411 [pii]; 10.1111/j.1365-2982.2009.01411.x [doi]. 10.1111/j.1365-2982.2009.01411.x 19775251

